# Cell Sources for Tissue Engineering Strategies to Treat Calcific Valve Disease

**DOI:** 10.3389/fcvm.2018.00155

**Published:** 2018-11-06

**Authors:** Eva Jover, Marco Fagnano, Gianni Angelini, Paolo Madeddu

**Affiliations:** Bristol Medical School (Translational Health Sciences), Bristol Heart Institute, University of Bristol, Bristol, United Kingdom

**Keywords:** valve heart disease, calcification, tissue engineering heart valves, *in vitro*, heterotopic bone formation

## Abstract

Cardiovascular calcification is an independent risk factor and an established predictor of adverse cardiovascular events. Despite concomitant factors leading to atherosclerosis and heart valve disease (VHD), the latter has been identified as an independent pathological entity. Calcific aortic valve stenosis is the most common form of VDH resulting of either congenital malformations or senile “degeneration.” About 2% of the population over 65 years is affected by aortic valve stenosis which represents a major cause of morbidity and mortality in the elderly. A multifactorial, complex and active heterotopic bone-like formation process, including extracellular matrix remodeling, osteogenesis and angiogenesis, drives heart valve “degeneration” and calcification, finally causing left ventricle outflow obstruction. Surgical heart valve replacement is the current therapeutic option for those patients diagnosed with severe VHD representing more than 20% of all cardiac surgeries nowadays. Tissue Engineering of Heart Valves (TEHV) is emerging as a valuable alternative for definitive treatment of VHD and promises to overcome either the chronic oral anticoagulation or the time-dependent deterioration and reintervention of current mechanical or biological prosthesis, respectively. Among the plethora of approaches and stablished techniques for TEHV, utilization of different cell sources may confer of additional properties, desirable and not, which need to be considered before moving from the bench to the bedside. This review aims to provide a critical appraisal of current knowledge about calcific VHD and to discuss the pros and cons of the main cell sources tested in studies addressing *in vitro* TEHV.

## Introduction

**Figure 1:**
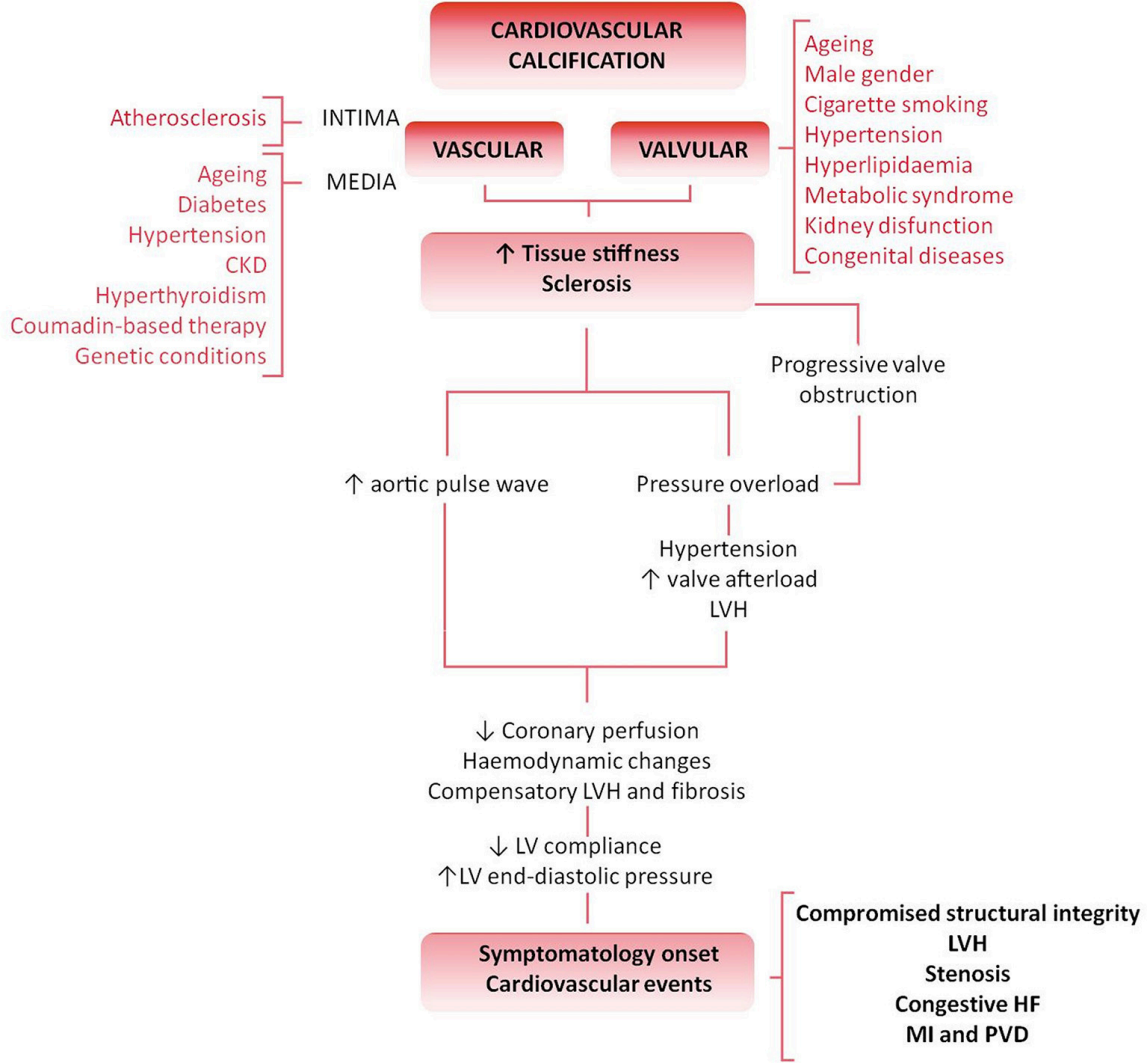
Differential pathology and clinical impact of valvular vs. vascular calcification flowchart. Cardiovascular calcification is an active and degenerative bone-like process affecting the cardiovascular tissues. Both vessels and valves show an athero-inflammatory background and, despite the commonalities and overlap of several risk factors (such as aging, hyperlipidaemia or kidney disease), both atherosclerosis and calcific VHD are two independent pathologic entities. The biological progression of the disease, tissue characteristics and clinical impact stand those differences. The result is the independent plaque rupture primary outcome found in the progression of VHD. An increased stiffness or sclerosis induces an increased aortic pulse wave, triggering hypertension, and a reduction in coronary perfusion. Besides, the pressure overload caused by a sclerotic pre-stadium and observed in the progression of the VHD leads to LV structural and hemodynamic changes. Symptomatology onset and calcification burden are poor prognosis predictors associated with multiple adverse cardiovascular complications, such as left ventricular hypertrophy (LVH), aortic valve stenosis, congestive heart failure (HF), ascending aorta aneurysm, myocardial infarction (MI), and peripheral vascular disease (PVD).

Cardiovascular calcification (CVC) is an independent risk factor and an established predictor of adverse and disabling cardiovascular events (Figure 1) (1–3). Histopathological studies have demonstrated hydroxyapatite deposits in vulnerable atherosclerotic plaques (4) and aortic valves (5). No longer considered a passive age-related disease, CVC is identified as the active, progressive and multifactorial ectopic bone-like calcification of blood vessels, myocardium or heart valves, leading to the “degeneration”/deterioration and dysfunction of the affected tissue (5, 6). Although there is an overlap between the risk factors leading to atherosclerosis and valvular calcification, only 40–50% of patients diagnosed with atherosclerosis concomitantly develop calcific valvular heart disease (VHD), thus suggesting that VHD is an independent pathological entity (7, 8).

VHD is the third most common cardiovascular pathology after hypertension and coronary artery disease in developed nations (9). Specifically, aortic valve stenosis (AVS) is the most common primary valvulopathy because of either congenital malformation (such as bicuspid aortic valve or BAV) or senile “degeneration.” The result is an increased stiffness and impaired leaflet motion and calcification, lately leading to the left ventricle outflow obstruction (10). Moreover, aging, male gender, cigarette smoking, hypertension, hyperlipidaemia, metabolic syndrome or kidney dysfunction are frequent independent risk factors for calcific VHD and significantly impair the outcome and prognosis of patients (11). The progression of calcific VHD consists of a valve sclerosis prestadium affecting more than 25% of the general population over 65 years old and associated to a 50% increased cardiovascular risk over 5 years (2). The prevalence of calcification and stenosis is reported in ~2% or 2.5% in a population aged over 65 or 75 years, respectively, representing a major cause of morbidity and mortality in the elderly (11, 12). Stenotic aortic valves are also found in congenital bicuspid valves and may require valve replacement even two decades earlier than valves anatomically normal (13). VHD is predicted to become a new cardiovascular epidemic in the next 20 years because of the increase of life expectancy in industrialized nations (9, 12, 14). No specific pharmacological strategy has been developed to retard, halt or revert the progression of VHD. Valve replacement represents the gold standard method to treat VHD through either mechanical or biological prosthesis implantation (15), but it is not suitable or definitive for all patients. New therapeutic solutions are claimed from the clinic to overcome the limitations of current therapeutic options including the chronic oral anticoagulation required for mechanical valves implantation or the degeneration, calcification, and failure of the biological counterparts. A plethora of novel tissue engineering-based approaches has emerged promising a definitive solution. Between the two main tissue engineered heart valves (TEHV) approaches, *in vitro* TEHV may provide, among others, a “native-like” extracellular matrix (ECM) surrogate and promote a “physiologic-like” regeneration in a pathologic environment with a deteriorated reparative system. Implantation of those devices is appealing for pediatric patients with congenital VHD as it might circumvent the failure of growth, repair, and remodeling required after somatic growth. In this review, we assess the current knowledge in the clinical relevance and mechanisms of valvular calcification and critically discuss the benefits and limitations of different cell sources currently used for the development of *in vitro* TEHV.

## Detection, risk and prevalence of valvular calcification

Calcific VHD of anatomically normal valves is a slow and active process driving to degeneration and dysfunction, with a long preclinical and asymptomatic phase. The onset of symptomatology is a general sign of advanced and severe disease associated with a high event rate, rapid valve deterioration and malfunctioning, thus being a poor prognostic indicator and elective for valve replacement surgery (15). However, the management of patients with asymptomatic valve disease is challenging. The real prevalence of unsuspected VHD is unsure, and a significant proportion of patients remain asymptomatic and undiagnosed until late stages when the long-term benefits of intervention are ambiguous due to increased postoperative complications and further mortality (8, 14). Large European and North American observational studies have provided most of the valuable insights on the overall VHD prevalence and the effect on overall survival (8, 14, 16, 17). In 2001, the Euro Heart Survey study (8) evidenced “degeneration” as the dominant etiological cause of VHD, with AVS (43%), mitral regurgitation (32%), and aortic regurgitation (13%) representing the commonest forms of adult valvopathies. AVS progression occurring in up to 5% of elderly patients (11, 14) carries an 80% 5-year risk of developing heart failure, valve replacement requirement, or death (18). Moreover, a US population-based study in more than 28,000 adults demonstrated the age-dependent VHD prevalence, rising from 0.7% in subjects aged 18–44 to 13% in those over 75 years old (16), significantly impacting the survival rates and emphasizing its significance as a health care issue. A more recent publication showed that general population aged ≥60 years across 37 advanced economies (16.1 million people) has a whole prevalence of 4.5% VHD (2.8 and 13.1% in individuals aged 60–74 and ≥75 years, respectively) (19). Only in the UK, VHD might account for approximately 1 million people aged over 65 years, and trend predictions suggest a significant raise due to increased life expectancy and the continuum of population aging in industrialized countries. The degeneration of anatomically normal valves is more often and rapid in people over 70 years because of progressive fibrosis and calcification of the valve cusps (www.bcs.com). A population aged over 75 years is projected to rise around 50% by 2025 resulting in a substantial VHD impact (www.statistics.gov.uk) recently estimated in ≈331,300 new cases of severe aortic stenosis per year including 65,600 patients (19). Thereby, VHD may become the next imminent and real cardiac epidemic (9, 12, 20). Genetic background and structural valve differences due to congenital malformations, such as BAV may be considered separately and are not deeply discussed in this review.

The presence and extent of CVC are generally acknowledged as strong predictors of future adverse clinical events including cardiovascular and all-cause mortality (21–23). The latter is highlighted by the up to 73% all-cause survival rate reduction estimated in patients diagnosed with high coronary artery calcification score (21). Importantly, 5–20% of the atherosclerotic lesions contain calcium deposits (24, 25), and it is alarming the potential underestimation of affected tissues due to the presence of chondrogenic intermediates, asymptomatic phases, or the lack of more powerful calcification screening methods. Additionally, the extent of valvular calcification correlates with the severity of stenosis (26). Therefore, a comprehensive and early understanding of the cardiovascular risk associated with calcification is critical for patient management and long-term prognosis (15, 27, 28).

Echocardiography is the mainstay for diagnosis, assessment and follow-up of VHD (15). It allows the calculation of the continuity equation-based aortic valve area both for predicting the clinical outcome and for clinical decisions making as well as aortic jet velocity and leaflet calcification (5, 29). However, visualizing abnormal valve anatomy becomes difficult once severe calcification is established. Moreover, concomitant hypertension increases the systemic vascular resistance in addition to the valvular obstruction, thus imposing a double over-load on the left ventricle which may lead to underestimate the assessment of the stenosis severity (30).

Other imaging methods, notably cardiac magnetic resonance imaging (MRI) and coronary computed tomography (CCT), are used if echocardiographic imaging is not satisfactory. Three-dimensional time-resolved, phase contrast cardiac magnetic resonance, otherwise referred as 4-dimensional (4D) flow MRI, is an innovative and appealing method for studying cardiovascular diseases. Dataset integration of 4D-Flow MRI can be retrospectively quantified providing a comprehensive evaluation of complex secondary vascular parameters, such as mechanical wall shear stress (WSS) on the vessels and heart valves (31) but also flow energy loss and flow displacements (32, 33). BAV is frequently associated with the progression of ascending thoracic aorta aneurysm (AsAo). Intrinsic wall abnormalities cannot fully explain the differential aneurysm progression resulting from different aortic leaflet fusion patterns and asymmetry (34). Echocardiography findings have suggested that abnormal blood flow could potentially trigger those differences in AsAo progression. In the context of VHD, 4D-Flow MRI has demonstrated to be a powerful tool to determine the association of flow hemodynamic, especially in those situations in which eccentric systolic blood flow jets result in abnormal helical systolic flow. The latter has highlighted the potential application of 4D-Flow MRI to study the progression and stratify/predict the risk of AsAo development specially in BAV patients (34), while echocardiography is not a reliable method. Moreover, recent studies have demonstrated the association of WSS and aortic peak velocity with parameters of left ventricle remodeling, allowing to distinguish BAV patients with or without aortic stenosis or regurgitation (35). Post-operative follow-up of reparative surgery of tetralogy of Fallot is another potential application of 4D-Flow MRI (36). However, long acquisition times, lack of blood pressure determination, susceptibility to motion artifacts, poor spatial resolution and the need of massive data post-processing are the main drawbacks of this technique. In addition, aberrant hemodynamic changes are seen only in advanced stenotic VHD and that represents a limitation for hemodynamic analysis techniques. Earlier phases of the VHD, such as asymptomatic sclerosis pre-stadium of well-functional anatomically normal valves, may not be detected by echocardiography or 4D-Flow MRI. Complementary imaging techniques such as CCT may provide substantial information on the detection and risk assessment of VHD. Multi-slice CCT together with the implementation of new acquisition techniques including ECG synchronization, retrospective image reconstruction and application of algorithms such as Agatston score (37), permit a direct, real-time and easy assessment of calcium content in coronary arteries (38). It has substantially improved the detection of early CVC stages. The high sensitivity of CCT has improved the screening for CVC, evidencing a progressive increment on CVC in patients over 60 years, which is especially relevant in patients diagnosed with VHD. Moreover, CCT combined with coronary angiography (gold-standard for coronary lesion evaluation) has demonstrated a good correlation between coronary calcium content and coronary artery disease (39, 40). The suitability of CCT to screen early stages of valve calcification in sclerotic valves with no hemodynamic obstruction has been also demonstrated (41). Moreover, CCT screening has proved to be a superior and more trustable method than carotid-intima-media thickness or ankle-brachial index for identifying patients at high risk (42). Finally, MRI and CCT can also provide complementary information to improve assessment of the valve lesion and cardiac function to aid the timing of surgery and determine risk (43).

## Pathophysiology of valvular calcification

**Figure 2:**
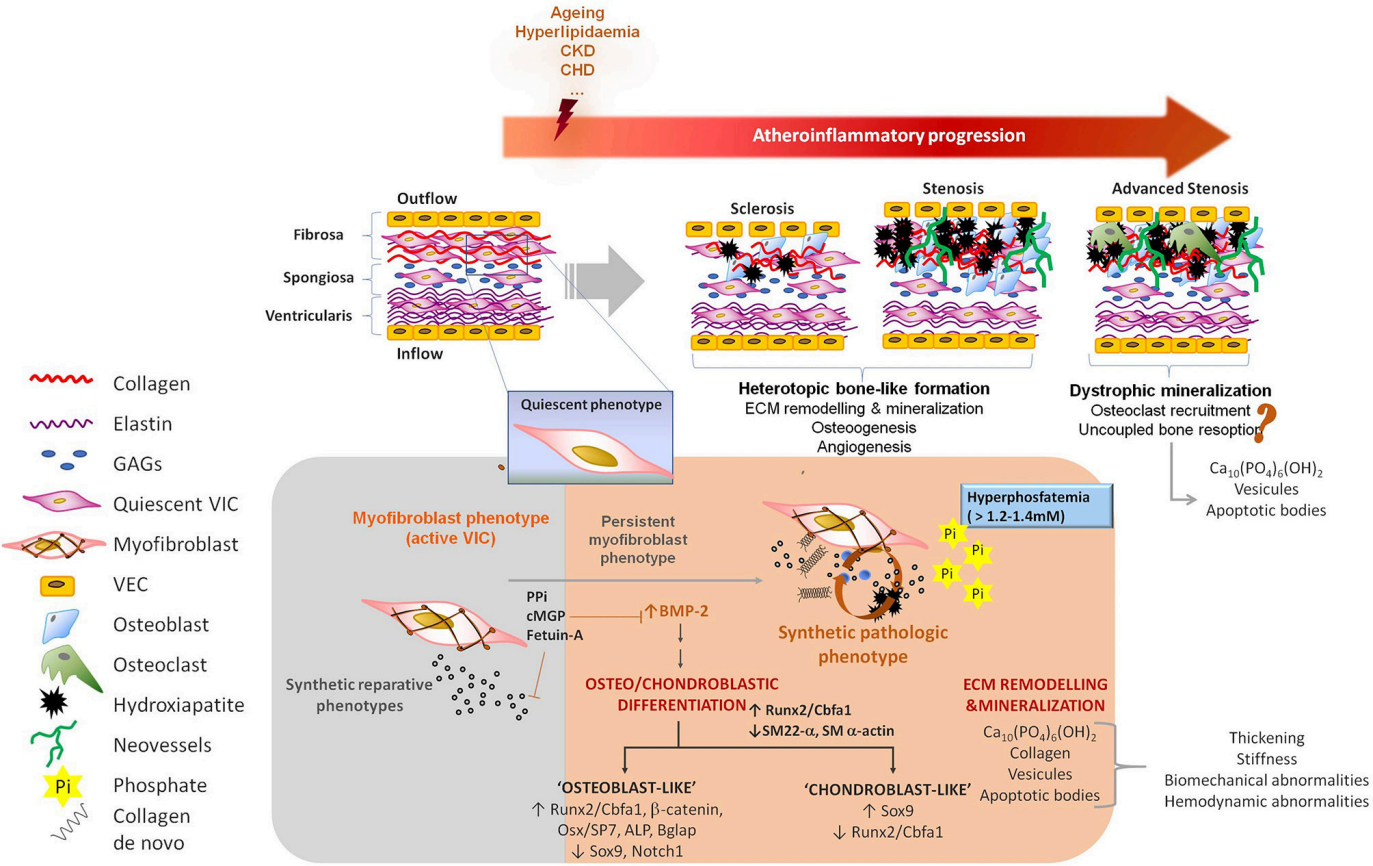
Pathophysiology of valve calcification. VICs are myofibroblast-like cells endowed with high plasticity, allowing them to participate in reparative, regenerative, and pathological processes. Underlying athero-inflammatory disease, aging as well as other clinical conditions such as chronic kidney disease or hyperlipidaemia, can trigger the acquisition of synthetic, proliferative and migratory phenotypes by VICs. These conditions are often associated with a reduction of defense mechanism against calcification. Two major features are recognized during valve calcification: (i) ECM remodeling, including the synthesis of a collagen-enriched matrix and its mineralization, and (ii) osteo/chondroblast differentiation of VICs. Osteogenic processes are associated with the abundant synthesis of collagen type-I and other ECM remodeling processes causing stiffness changes capable of perpetuate and extend the osteogenesis in the valve. A prestadium of sclerosis in well-functional valves finally leading to stenosis and obstruction in an active heterotopic bone-like formation process has been described. Observations in human samples also suggest the possibility of dystrophic mineralisation in advanced end-stages of calcific VHD. Pyrophosphate, osteopontin, osteoprotegerin, gamma-carboxylated matrix Gla protein and circulating fetuin-A are listed as physiologic inhibitors of vascular calcification; while hyperphosphatemia and hypercalcemia, expression of bone morphogenetic proteins or alkaline phosphatase and activation of osteo/chondroblast regulators (Runx2, Osterix, Wnt) are counted among the pro-calcifying elements orchestrating osteoblast-like transdifferentiation. PPi, pyrophosphate; cMGP, gamma-carboxylated matrix Gla protein; BMP-2, bone morphogenetic protein 2; ECM, extracellular matrix; CKD, chronic kidney disease; CHD, congestive heart disease; VIC, valve interstitial cells; VEC, valve endothelial cells; GAGs, glycosaminoglycans; Ca_10_(PO_4_)_6_(OH)_2_, hydroxyapatite; Pi, inorganic phosphate; ALP, alkaline phosphatase; Bglap, bone Gla protein or osteocalcin.

Over the past four decades, experimental and clinical research has elucidated the pathophysiology of CVC. Ectopic calcification is an active and tightly organized process, which recapitulates several molecular mechanisms orchestrating physiologic chondro/osteogenesis (44–46). Both the phenotypic trans-differentiation into osteo/chondroblast-like cells and the active ECM remodeling, including its mineralization, represent the two hallmarks of ectopic calcifications (47) (Figure 2). In particular, calcific VHD has been described as a multifactorial, complex and active heterotopic endochondral lamellar bone-like formation process, driving heart valve calcification, degeneration and dysfunction (5, 6) toward integration of ECM remodeling, osteogenesis, and angiogenesis. Heterotopic bone exhibits morphological and biochemical features of orthotopic bone, and it is capable of generating bone marrow (48). Higher remodeling rates have been reported in calcified valves than in physiologic bone formation (48) though, thus suggesting uncoupling of bone formation and resorption activities (49). However, histological observations in human specimens of calcific valves have evidenced an 83% prevalence of dystrophic calcification with only a 13% of active bone remodeling (5) and a 92% prevalence of microfractures, which are the main site of active bone remodeling. One could interpret valve calcification as a senile degeneration leading to cellular aging and death, and hydroxyapatite deposition on cellular degradation products rather than an active osteogenic-derived mineralization occurring on collagen and elastin fibers. Further research on this regard will significantly contribute to the understanding of the calcific VHD pathophysiology in the next years and it is currently cause of controversy. Observation of advanced end-stage phases in dystrophic human specimens could lead to misinterpretation of the underlying pathology in which a “preliminary” heterotopic bone-like tissue demonstrated by both *in vitro* and *in vivo* studies, might lately be replaced toward a misbalanced bone resorption (49) leading to an increased presence of dystrophic mineralisation.

Mature valves have an avascular (50) trilaminar organization including the upper surface of the valve or fibrosa (outflow), the central spongiosa and the inflow-orientated ventricularis. Those layers differ with each other by distinct ECM organization, composition and mechanical properties [a detailed description of the valve anatomy and function has been provided by Schoen (44)]. Moreover, two major resident cells are found in the valve: valve endothelial cells (VEC) and valve interstitial cells (VIC). Although VECs are not fully characterized, differential phenotypes and expressional profiles have been identified (51, 52). Biological functions of the VEC may include regulation of permeability, mediate immune responses and establish a paracrine signaling with VICs (53). The VIC represents a heterogenous population of mesenchymal cell type which shares several commonalities with vascular smooth muscle cells (VSMC) and fibroblasts, acts as mechanical sensor thorough complex cell-to-ECM interactions and shows highly dynamic phenotype plasticity (54). The latter allows the VIC to contribute to the permanent ECM turnover and reparative processes guarantying the maintenance of the valve integrity and function (44), but also contributes to the development of valve stenosis (55, 56).

Calcific VHD is regarded as an active athero-inflammatory disease associated with a damaged endothelium and an unresolved immunological inflammation resulting from such insult. The pathogenic role of hyperlipidaemia in the valve was recognized toward the introduction of dyslipidaemia experimental models (57, 58). Early studies in human aortic valve lesions demonstrated the association among atherogenic oxidized low-density lipoproteins (oxLDL) risk factor and the expression of signaling molecules promoting osteogenic processes (57). Moreover, inflammation plays a key role on the pathogenesis of VHD with superimposed calcification (59). Numerous histological studies have suggested inflammation to trigger ECM remodeling, fibrosis, and valve thickening leading to the structural changes of VHD (60) and the subsequent differentiation of VICs into osteoblast-like phenotypes (55). Despite the commonalities and overlap of several risk factors, both atherosclerosis and calcific VHD are currently considered two independent pathologic entities mainly due to differences in the biological progression of the disease, tissue characteristics, clinical impact and resulting outcomes independent of plaque rupture (8). Among other differences, a CD8 T cell-based inflammatory background has been evidenced in valve calcification instead of the polyclonal lymphocytes reported in atherosclerotic lesions (59, 61).

The discovery of genetic modifications such as Notch1 mutations and their association with dysfunctional tissue structure of BAV and the spectrum of VHD has increased the current knowledge of these abnormalities through congenital cardiology (62). Signaling components of embryonic valvular development, such as Notch1 as well as bone morphogenetic protein (Bmp) members, transforming growth factor beta 1 (TGF-β1) or Wnt/β-catenin participate in the onset of AVS by contributing to ECM remodeling, osteogenesis and angiogenesis [further reviewed in (47, 63, 64)].

## Osteogenesis

Several cell types have been involved in the development and progression of CVC and that includes vascular resident cells (VSMCs, VICs, or VECs) and circulating cells, such as mesenchymal stromal cells (MSCs), endothelial progenitor cells (EPCs) or calcifying vascular cells (CVC) (47). A dysfunctional valvular endothelium together with an imbalance between activators and natural inhibitors may promote the calcification of neighboring VICs (Figure 2) (65–67). Different VICs sub-populations have been identified in the heart valve and may differentially contribute to the pathology of calcific VHD (54). Multiple signaling molecules (such as Bmp2, TGF-b, Wnt/b-catenin, VEGF, or Notch1) are integrated in what resembles a pathologic post-natal recapitulation of fetal valvulogenesis, including the acquisition of quiescent-to-active phenotypes, an active ECM remodeling, cytokine release and promoting *in situ* osteoblast/chondroblast-like differentiation as an environmentally maladaptation (68, 69). Bmp2 is a strong morphogen inducing osteoblast-like phenotypes, and it plays a key role in the pathogenesis of VHD (47). Bmp2 signaling triggers nuclear translocation of Smad proteins and the activation of osteogenic-regulators such as Runx2/Cbfa1. Runx2 is an early master gene of osteoblast differentiation and chondroblast maturation during heterotopic endochondral bone formation (5, 6, 70). Active Runx2 can bind to the SP7 promoter to induce the expression of Osterix, a master transcription factor of differentiated osteoblasts. Both Runx2 and Osterix bind to BGLAP promoter to induce osteocalcin, a marker for differentiated osteoblasts, which contributes to maturation of the mineralised ECM and it is present in calcified heart valves (69). Moreover, ECM synthesis is induced directly by Osterix and its binding to the *COL1A1* promoter or indirectly by Runx2 through the activation of *ATF6* and subsequent *COL1A1, COL1A2*, and *BGLAP* expression (66, 67, 70). In addition, Osterix triggers the expression of alkaline phosphatase (ALP), a pyrophosphatase capable of releasing inorganic phosphate from PPi, thus inducing local hyperphosphatemia and PPi deprivation. ALP also inhibits osteopontin phosphorylation and thus its protective biological function. Finally, Wnt/β-catenin pathway mainly perpetuates osteoblastic phenotype by further induction of Runx2 and Osterix toward Bmp2-dependent signaling (57).

It is now appreciated that VECs, under certain circumstances, may undergo endothelial-to-mesenchymal transition, which is reminiscent of the early formation of the endocardial cushions (71, 72). The result might be an increase in the number of VICs susceptible to display an osteoblast phenotype (45). Dysfunctional VECs also manifest an altered secretome (73). VEC-derived nitric oxide (NO) is a regulator of Notch1 signaling in calcifying VICs (74), and a decreased Notch signaling has been found in AVC (45). Moreover, genetic studies in BAV have identified eNOS and Notch1 as candidate genes contributing to the valve anatomy and the development of VHD (62). Notch signaling leads to the cleavage and nuclear internalization of the Notch1 intracellular domain. One of the target genes of the Notch1 intracellular domain is the Hairy/enhancer-of-split (Hes)-related with YPRW motif (Hey) element, which is involved in early valve development. Both the nuclear location of the Notch1 intracellular domain and the expression of Hey1 are regulated by the VEC-derived NO (74). Furthermore, Notch1-dependent signaling is transduced through Bmp2/Runx2 axis, which directly regulates Sox9 in chondrogenesis and is an important mediator of AVS (75). Hey1 activated by Notch1 signaling forms a complex with Runx2/Cbfa1 and inhibits its transcriptional activation (64).

Pluripotent resident cells, EPCs, MSCs, and MSC-like pericytes have been found in calcified lesions suggesting a role of progenitor cells in the development and progression of ectopic calcification (45, 76, 77). The athero-inflammation associated with the release of multiple cytokines and chemokines may contribute to the recruitment of stem/progenitor cells into an environment whose homeostasis has been hampered by pro-calcifying factors and the depletion of physiologic calcification inhibitors. Finally, bone marrow (BM)-derived cells may contribute to replenishing the VIC population, modifying the proportion of VIC subpopulations yielding increased susceptibility to calcification. By using chimeric mice whose BM was repopulated with enhanced green fluorescent protein expressing total nucleated BM cells, Hajdu et al. documented the engraftment of BM-derived cells occurs as part of normal valve homeostasis (78).

### ECM remodeling

Besides providing biomechanical support, valvular ECM participates in a plethora of biological functions, such as cell communication and differentiation. In addition, the ECM may contribute to ectopic CVC (74, 79, 80). Differentiation of VICs toward myofibroblast or osteoblast phenotype is highly dependent on the complex and unique VIC-to-ECM components interactions (81). Therefore, a loss of the valve ECM integrity causes malfunction and results in VHD. In line with this, propagation of the inflammation-dependent calcification of the heart valves is associated with the active ECM remodeling resulting from the proteolytic and synthetic activities of active macrophages, VICs and mast cells (82). Moreover, substrate stiffness elicits the myofibroblast activation of VICs which remaining persistent can lead to osteoblast differentiation although the exact molecular mechanisms remain unclear (83, 84).

Regulatory factors, such as thrombospondins, have been found characteristically up-regulated in calcific valves (47). Moreover, microstructural changes in collagen fiber number, width, length, density or alignment may regulate pathogenic processes compromising the mechanical properties of the valve, in particular, and most frequently at the level of the spongiosa, chondrogenic-like layer (80). Mechanistically, cartilage-specific ECM genes are downregulated in calcifying VICs because of Sox9 downregulation (74). Moreover, ECM influences VEC function and it is involved in VEC-to-myofibroblast transformation toward EMT processes (85).

### Angiogenesis

Heart valves have a sparse vascularity at the proximal part (50) being considered mainly avascular. That valve avascularity is seemly abrogated in VHD (86), and the extent of neovascularisation correlates well with the burden of the disease (87). The expression of pro- and anti-angiogenic factors in stenotic valves or calcifying VICs (74, 88) has reinforced the idea that angiogenesis in the valve may promote calcific VHD, which calls for the use of modulators of angiogenesis in the therapy of valve degeneration (86, 88, 89). Accordingly, anti-angiogenic therapy has shown a protective effect on the valvular cusp endothelium (86). Immunohistochemical studies have revealed the co-localization of micro-vascularization with actively proliferating VICs, bone-related proteins, and heavy calcification (90). In addition, during calcific VHD, expression of osteonectin (pro-angiogenic and chondrogenic factor) and Lect1/chondromodulin-1 (Chm-I) (anti-angiogenic factor) is disrupted (74). In human calcified valves, the expression of vascular endothelial growth factor (VEGF), matrix metalloproteinases (MMPs) and angiogenesis is concomitant with a downregulated expression of Chm-I.

Revisiting the physiology of bone formation, one could speculate that valve angiogenesis is not the cause, but the consequence of the osteogenesis perpetuation described for endochondral bone formation or that may provide of support to the thickened tissue produced *de novo* and resulting in a hypoxic microenvironment requiring of oxygen supply (50). An angiogenic switch of cartilage allows neovascular invasion and triggers the replacement of cartilage by bone (91). Resting chondrocytes become active and proliferative to differentiate into pre-hypertrophic chondrocytes, which can then secrete the cartilage matrix (92). Angiogenic factors such as TGF-β or VEGF are normally expressed in the cartilage and Chm-I has been proposed as an inhibitor during avascular phases of chondrogenesis. Chm-I plays divergent biological functions including chondrocyte growth and angiogenesis inhibition, stimulation of osteoblast proliferation and differentiation with a reduction of ALP activity (92, 93) and contribution to bone remodeling (92). Accordingly, Chm-I expression is upregulated by Sox9 during chondrogenesis in the avascular cartilage, but it is not present in the late hypertrophic and calcified zones leading to final osteogenesis (88, 91). In line with this, Chm-I deficient mice showed a significant increase in bone mineral density and lowered resorption (92). Moreover, the basal cartilage-like profile of the normal and mature valve is lost during AVS, though *in vitro* assays have shown an early up-regulation of Sox9 followed by Runx2 and ALP up-regulation (94). This may indicate an early chondroblast intermediate stage before the down-regulation of Sox9 and Cmh-I. Noteworthy, angiogenic factors and abundant vascularization have been mostly co-localized in late-stage heavy calcified plaques (87, 94) with the presence of osteopontin and osteocalcin suggesting a mature mineralised ECM (90, 95) but also coinciding with the thickest remodeled tissue. Importantly, VEGF induces osteoblast proliferation and differentiation and osteoclast recruitment (96) but also inhibits calcification of ovine VICs in the presence of particular ECM compositions (97), highlighting again the regulatory importance of the ECM in modulating the action of growth factors.

According to another theory, VHD recapitulates the signaling pathways that control developmental valvulogenesis (98). For instance, Bmp2, canonical Wnt, TGF-β1, and Notch signaling occurs during the endothelial-to-mesenchymal transition (99, 100) which may induce a myofibroblast phenotype on VECs and the subsequent calcification if the signaling network activation persists in time.

## Current therapeutic approaches for calcific aortic valve stenosis

Several pharmacological attempts have been made for establishing a medical treatment of CVC. The regression observed by *in vivo* calcification models suggests the existence of endogenous mechanisms capable of dismantling the extremely insoluble and stable calcium phosphate deposits (101). Potential strategies to revert CVC have been proposed during the last few years and reviewed by O'Neill et al. (101). Preliminary evidence suggests a beneficial effect of treating calcific VHD, but frequently in association with bone mass weakening (101). Therefore, preventing or reverting the ectopic bone-like formation in the cardiovascular territory may boost bone resorption and increase the risk of fractures, which represents a serious concern for extensive use in the elderly population. To date, surgical valve replacement (SVR) represents the only available therapeutic approach for treating VHD.

Valve replacement, specifically aortic valve replacement, represents the second commonest cardiovascular surgical procedure (102) and accounts for 10 to 20% of all cardiac surgical procedures in the US (9). A 26% increase in the number of patients undergoing aortic SVR was calculated over a 5-year period comprising 2004–2009 in Great Britain and Ireland (103). It is anticipated that the number of patients requiring SVR will be 2.93-fold increased by 2050, in less than 50 years' time. Refusing to undergo SVR is associated with poor prognosis, a significant morbidity (104, 105) and >12-fold the risk of mortality (105). More than half of the patients will die within the next 12–18 months of symptom onset (106). Risk factors, co-morbidities and patient denial are common exclusion criteria for valve replacement. According to a recent survey, about 40% of patients with severe symptomatic VHD and 70% of patients with asymptomatic VHD were not eligible for SVR (19). This heterogeneous population require therefore alternative approaches.

The introduction of SVR has improved the outcome of patients with VHD. Mechanical or biological prosthesis are the two main options for current SVR (107). Mechanical valves last longer and are still the gold standard for patients under 60 years (108), but may come with a high inherent risk of thrombosis and therefore a requirement for chronic oral anticoagulation, based on coumadin derivatives. More frequent use of biological prostheses (mainly porcine or bovine-derived), introduction of minimally-invasive implantation techniques, and better control of risk factors and complications (109, 110) have considerably improved the clinical outcome of people undergoing SVR (111). Geometrical, nano-structural and material features of the bioprosthetic valve are more similar to the native tissue. Moreover, recent bioprosthetic valve improvements have significantly lowered the age for recommended mechanical valve replacement (111, 112). Nevertheless, biological prosthesis has a relatively poor long-term durability; thus, it does not provide a definitive cure. Instead, owing to the progressive deterioration and failure of current valve substitutes, native VHD is traded for “*bioprosthetic valve disease*,” which entails expensive treatments, hospital readmission and reintervention (110). Structural prosthetic valve deterioration represents a major limit for durability, independently the substitute is a homograft or xenograft. Several factors contribute to this phenomenon. Animal-derived prostheses, now prevalently used because of the shortage of human valve substitutes, are subjected to decellularization procedures to prevent recipient's immune response, and are cross-linked with glutaraldehyde, to provide tensile strength and elasticity, and render them further non-immunogenic. However, improvements in pliability and tolerogenicity come at a price. In fact, elimination of VICs, which synthetize ECM proteins and possess contractile properties, deprive the valves of their unique function in such a mechanically demanding environment and makes prostheses more susceptible to degeneration. Moreover, residual fragments of devitalized VICs and VECs may act as hydroxyapatite nucleation sites and induce activation of immune responses. Atherosclerotic processes also participate in prosthetic valve remodeling, with initial accumulation of oxLDL, followed by monocyte recruitment, generation of a pro-inflammatory milieu, collagen disruption and osteogenic differentiation of resident endothelial cells (ECs) and precursor cells recruited from the circulation (113, 114). Damage of the ECM is cumulative: calcium deposits enlarge and merge, forming nodules that interfere with the bioprosthesis function. Manufacture protocols preserving ECM integrity and encouraging *in vivo* recellularization prolong durability (115, 116). Anti-calcifying agents are also effective (117). However, ECM disorganization and degradation remains the ultimate limiting factor in durability (118).

Pediatric or adolescent patients diagnosed with congenital valve diseases are specially challenging. The risk of prosthetic valve failure becomes relevant in these populations with a 10% rate of failure within 4 years after implantation (117) and usage of mechanical valves linked to chronic oral anticoagulation does not fit with their active lifestyle. Failure of somatic growth, repair and remodeling are also common problems of both mechanical and biological prosthesis. The ideal valve prosthesis has yet to be developed.

## Future solutions from regenerative medicine and tissue engineering of heart valves

Landmark experimental and clinical work has demonstrated the potential of tissue engineering, which combines cells from the body with template materials, to guide the somatic growth of new tissue and correction of organ defects (119). Application of this approach has been proposed to improve the durability of cardiac prostheses and thereby optimize long-term outcomes in patients with congenital or acquired valve defects. Therefore, Tissue Engineering of Heart Valves (TEHV) has emerged as a valuable alternative for definitive treatment of VHD promising to overcome either the chronic oral anticoagulation or the time-dependent deterioration and reintervention of current mechanical or biological prosthesis, respectively and to offer a valve substitute capable to grow in a “physiologic-like” manner. In the past few decades, two main strategies have been developed to generate TEHVs. The underpinning concept for both TEHVs approaches is that patient's own cells will generate a viable and physiologically competent tissue able to withstand hemodynamic forces before (*in vitro*) or after (*in situ*) implantation. Briefly, *in vitro* TEHV uses various types of autologous cells, including stem/progenitor cells, that are expanded in culture, seeded on decellularized biological (120, 121) or synthetic scaffolds (122, 123) (see below), and may be conditioned in a bioreactor to ensure fast and competent “natural-like” matrix production before implantation (124). The underlying concept is that *in vitro* incorporation of cells shall confer prosthetic grafts with the characteristics of a living tissue that remodel in a physiologic manner and concert with cardiac and whole-body needs, withstanding the impact of degeneration and calcification. Implantation of *in vitro* TEHV is an appealing alternative for pediatric patients with congenital VHD requiring of SVR even two decades earlier than VHD patients with anatomically normal valves. On the other hand, i*n situ* TEHV, aims to create an acellular biodegradable scaffold which gradually transforms into a living valve by recruiting endogenous cells upon orthotopic implantation (125–127). An interesting combination of the *in vitro* and *in situ* approaches is represented by tissue-engineered matrixes (TEMs), which are usually made of autologous vascular cell- or fibroblast-derived ECM/fibrin gel sheets undergoing a decellularization process before implantation. TEMs are supposed to provide a more natural substrate for homing of the recipient's cells (128, 129). A similar strategy to produce a natural ECM graft is the *in vivo* TEHV by which synthetic non-degradable molds are implanted at sub-cutaneous level and expected to produce a collagen-rich, non-immunogenic, harvestable, and implantable fibrotic capsule (108). In the *in vitro* procedures and TEM, a balance between the extent of decellularization and conservation of the native properties of the ECM must be reached to avoid undesired alterations of biomechanical and hydrodynamic properties. The *in situ* approach is instead totally reliant on the endogenous capacity of the hosting organism to mobilize and incorporate the *right cells*, which may be negated by a disease-associated alteration in cell behavior (130–133). The two main strategies, *in vitro* vs. *in situ*, are briefly summarized in Table 1.

**Table 1:** Advantages and disadvantages of *in vitro* and *in situ* TEHV.

	***In vitro* TEHV**	***In situ* TEHV**
Advantages	•Exogenous delivery of stem/progenitor cells in an environment with a deteriorated endogenous reparative/regenerative system	•More rapid implantation and possibly non-invasive
	•Promotion of a “physiologic-like” reparation/regeneration of the injured area by the exogenously delivered stem/progenitor cells	•Resident cell recruitment
	•Promotion of resident cell recruitment	•Surrogates mimicking the native ECM
	•Phenotype modification of recruited cells through stem/progenitor cell-derived secretome or other mechanisms	•Off-the-shelf scaffold manufacturing. Limitless supply and ready-to-be implanted for urgent implantations
	•Bioprosthetic-derived *in vitro* TEHV displays a more “native-like” ECM. Alternatives to GA cross-linking may inhibit calcification and there is a lower ECM damage than in decellularized tissue used in many HV implants	•Different and desired growth factor or drugs can be delivered
	•A dynamic maturation prior implantation may favor a desired cell profile and ECM remodeling, including collagens as well as non-collagen proteins (e.g., proteoglycans and glycosaminoglycans)	•Tissue Engineered Matrix (TEM)-*in vitro* synthesis of a natural-like ECM
	•Cell seeding prior implantation reinforce the capability of the TEHV to support cell functions such as viability, proliferation or migration in both static and dynamic conditions	•Mechanical, chemical, and biochemical features of the construct will stimulate and direct the host's native regeneration capabilities
	•Inhibition of thrombogenic events	•Controlled and tailored properties
	•Tailored prosthesis according to patient's anatomy	•Easy, reproducible and less expensive formulation
	•Maintained natural ECM architecture and depending cell signaling	•Less prone to infections or contaminations
	•Biodegradable surrogates mimicking the native ECM	
	•Possibility of Tissue Engineered Matrix (TEM) application	
Disadvantages	•Time-consuming, need of cell harvest, expansion, repeated manipulation, potential infection	•TEM limitations-decellularization (and cross-linked) product, related toxicity and creation of calcium nucleation sites. Some could be not cross-linked
	•Decellularized products, related toxicity and calcium nucleation sites mainly if using GA cross-linking	•Lower capability of stem/progenitor reparative cell recruitment due to underlying impaired mobilization: physiologic reparative and regenerative processes must be lower under certain clinical conditions causing VHD
	•Multilineage commitment of exogenous stem/progenitor cells can favor undesired phenotypes	•Thrombogenicity in collagen-based exposed surfaces needing of rapid *in vivo* re-endothelisation in hypercoagulable diseases
	•Undesired phenotypes in recruited cells including myofibroblast profiles. Leaflet contraction	•Limited capability to modify diseased phenotype of recruited resident cells, mostly subjected to the physical properties of the scaffold.
		•Myofibroblast phenotype activation and leaflet contraction
	•Possible immunotherapy for allogenic cells. Autologous cells are not ideal for old patients or patients with CVD	•Time-limited delivery of drugs or growth factors
	•Product heterogeneity depending on baseline characteristic of the donor	•Prosthesis-patient mismatch in off-the-shelf products
	•Potential malignant transformation of derived cells	•Toxicity of degradation products. Induction of inflammatory response
	•Immunogenic response if decellularization process not completed •Optimal cell type/s to be determined •Advanced Therapy Medicinal Product-GMP regulations for cell product and scaffold may make more complicate to transfer the results from the bench to the bedside •Potential modification of prosthesis geometry in tailored prosthesis	•Difficult balance among hydrolytic polymer degradation and tissue formation in a systemic pathological environment which can drastically modify mechanical and biochemical properties

*In green color are highlighted the potential advantages; in red color the potential disadvantages. HV, heart valve; ECM, extracellular matrix; GA, glutaraldehyde; TEHV, Tissue Engineered Heart Valve; GMP, good manufacturing practice; CVD, cardiovascular disease*.

A three-dimensional scaffold and the correct choice of cells are the cornerstone elements to consider generating a living valve substitute. A plethora of approaches and techniques have been established on TEHV and that has been recently and extensively reviewed elsewhere (134–136). Unique valve mechanobiology features and implications in the development and design of TEHV has been nicely reviewed by Schoen (137). Furthermore, utilization of different cell sources may confer of additional properties to the valve substitute which may, or may not, be desirable in the VHD environment. The cell of choice to be seeded in *in vitro* TEHV should sense and perform optimal adaptative responses to environmental changes. All that must be considered before moving from the bench to the bedside and is further discussed below.

## Scaffolds for *in vitro* TEHV

Intuitively, the best scaffold/graft to comply with all the requirements of TEHV would be the native aortic valve-derived ECM or similar biological composites. Commercially available bioprostheses are being currently tested as cell carriers thanks to significant improvements in decellularization protocols, which include novel cross-linking procedures to increase pliability while avoiding calcification (138). For instance, glutaraldehyde fixation replaced by heparin has shown to ameliorate valve prosthetic calcification rates probably by blocking calcium phospholipid binding sites as well as to inhibit thrombosis (108, 139). Other cross-linking alternatives are the reduction of free amine groups and targeting free aldehydes by using reducing agents capable of forming Schiff bases which may allow for glycosaminoglicans and elastin stabilization, avoid collagen deformation and inhibition of calcium binding (138).

**Figure 3:**
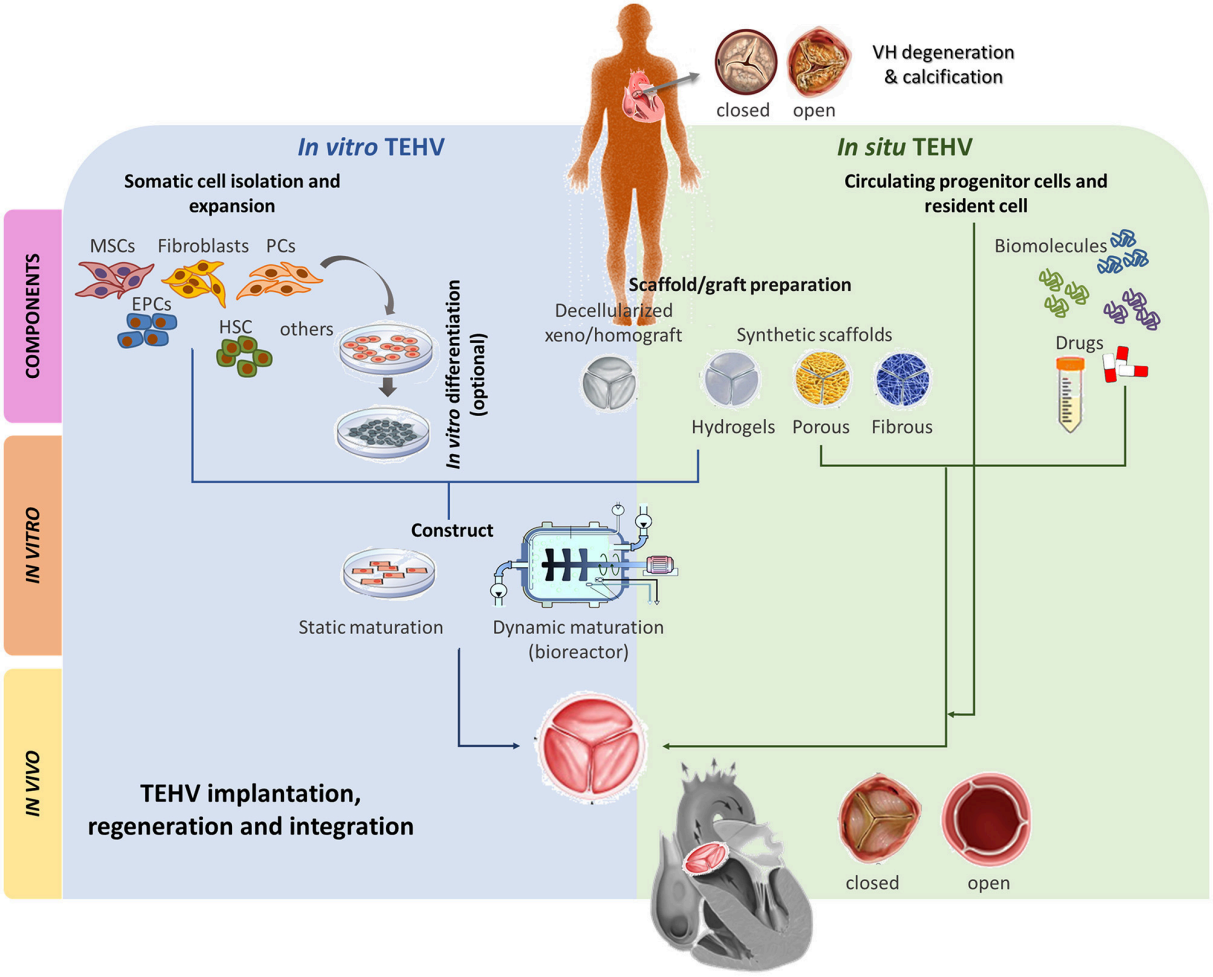
Main TEHV approaches comparison. Two main TEHV strategies are being explored; *in vitro* and *in situ. In vitro* TEHV consist of the combination of, ideally, autologous cells (e.g., MSCs, fibroblasts, EPCs, HSC, PCs or others) that may, or may not, be previously differentiated into the target cell. Those cells are seeded on a scaffold or graft preparation and statically or dynamically matured prior orthotopic implantation. On the other hand, *in situ* TEHV is based on the use of an acellular scaffold/graft capable to recruit endogenous cells which will remodel and integrate. Both *in vitro* and *in situ* meet at the scaffold/graft preparation step. Scaffold/grafts can be based on decellularized xeno/homografts or synthetic totally/partially biodegradable materials (hydrogels, porous, or fibrous). Both approaches can be enriched with biomolecules or drugs stimulating desired cell recruitment and phenotypes or inhibiting deleterious events such as calcification, angiogenesis, etc of the TEHV. After implantation, the TEHV must be able to regenerate and integrate into the recipient host and be native-like functional. MSCs, mesenchymal stromal cells; EPCs, endothelial progenitor cells; HSC, hematopoietic stem cells; PCs, pericytes.

As anticipated above, the typical *in vitro* approach is to seed cells on a scaffold, and induce differentiation and ECM synthesis. However, it has been demonstrated that respiring cells on the scaffold periphery and the size of the scaffold can restrict oxygen and nutrients availability at the center of the tissue, leading to areas of necrosis and degeneration(140). Different procedures have been proposed to circumvent this problem, including mechanical compression (141) and flow perfusion (142). The implementation of dynamic systems, such as use of bioreactors, before implantation into the recipient host may give better results than static systems and considerably contribute to maintain viability of the three-dimensional TEHVs supporting its maturation. Mature grafts/scaffolds might be easier to integrate into the recipient's heart and to acquire the definitive native features of a living valve (143–145) (Figure 3). However, dynamic culture conditions can also negatively impact cell differentiation and tissue formation. Since two intermediates products are combined in the final Investigational Medicinal Product [definition provided in *Directive 2001/20/EC, Article 2 (d)*], it is vital the latter is checked for quality and quantity before implantation.

### Cell types for *in vitro* TEHV

Cells represent the biological component of the TEHV, e.g., the ones supposed to confer living properties. The optimal cell type for valve engineering should be non-immunogenic and able to maintain its specialized function or gain such a specialization through differentiation. Autologous cells are the first choice, but they show significant dysfunctions if obtained from old patients or patients with cardiovascular diseases (146), while allogenic cells might be immunogenic (147). Induced pluripotent stem cells (iPSCs) generated by reprogramming somatic cells would be the ultimate solution for patient-tailored therapy but there are still several concerns (148). Hence, differentiated cells or progenitor cells, including VICs, VECs, MSCs, BM-mononuclear cells (MNCs), fibroblasts or EPCs, remain a safer option thus far. We will go through some examples (144, 149–155) here which are further summarized in Table 2.

**Table 2:** Different cell type candidates for *in vitro* TEHV.

**Cell type**	**Scaffold type**	**Experiment stages**	**Therapeutic activity**	**Outcome**	**Reference**
**MSCs** •Autologous BM-MSCs	•Nonwoven PGA/PLLA blend melt extruded into sheets assembled to valvular shape	•*In-vitro* dynamic for 4 weeks •*in-vivo* autologous in sheep up to 8 months	•Histology resembles the native tissue: elastin on the ventricularis side and collage on the fibrosa side •vimentin expressed throughout the scaffold as native tissue •α-SMA expressed only in subendothelial layer (as native t) •Continuous layer of vWF+ cells on the surfaces	•Closer to native tissue stiffness after 4 weeks. Burst resistance higher than native tissue •Extensive remodeling *in-vivo*, layering and differentiation •MSCs phenotype expression and synthesis mirrored the native tissue	(156)
Autologous BM-MSCs cultured in two different media: •EGM-2 for ECs differentiation •M199 for myofibroblast differentiation	•Decellularized porcine pulmonary valves	•*In-vitro* static •*in-vivo* canine model up to 1 week (pulmonary position) and 3 weeks (aortic position)	•CD31+ layer on the leaflets surface •Sporadic presence of α-SMA+ cells in the interstitial region	•Leaflets were intact, with no evidence of thrombus formation. Cellular coverage of leaflet surface and interstitial region repopulation	(153)
•Juvenile sheep BM-MSCs (α-SMA and vimentin +)	•Nonwoven PGA:PLLA (50:50) scaffold	•*In-vitro* static up to 4 days •*In-vitro* pulsatile flow with flexure stimulus (combined or not) up to 3 weeks	•Weak fibronectin and collagen I expression, high collagen III •Higher expression of α-SMA, CD31, vWF, and laminin (ECs markers apart α-SMA) on the surface compared with interstitium •Significant decrease of DNA content compared to native tissue •Higher DNA content in the flexure-flow sample than in the other ones •Higher S-GAG content in flex-flow samples	•Tissue formation and cellularity was higher in flex-flow samples by three weeks •Confirmed positive synergy of flexure and flow stimuli by SEM, H&E, DNA and collagen content assay •ECs markers might be the cause of a reduced collagen/cell ratio in the flex-flow samples •Phenotype plasticity of MSCs	(157)
•Non-selected lamb BM-MNCs •Non-selected lamb BM-MSCs	•Decellularized porcine pulmonary valves	•*In-vivo* in lambs up to 4 months	•In both groups, complete layer of VWF+, and α-actin+ cells demonstrated the presence of a thin ridge of SMCs, more pronounced in the MSC group •In adventitia and media of BM-MNCs group the ECM was disorganized with a strong macrophages infiltration (CD68); in adventitia many neo-vessels were present •In adventitia of BM-MSCs group the global organization of collagen fibers was preserved, rare neo-vessels were visible •No inflammatory cells were found in MSC group	•No significant pulmonary regurgitation was recorded at any timepoint •BM-MNCs group: wall thickening, calcifications, fibrous pannus covering the suture line; leaflets slightly thickened and retracted •BM-MSCs group: no calcifications, wall remained thin and smooth, no fibrotic pannus on the suture lines, thin leaflets	(154)
•Autologous sheep BM-MSCs	•Nonwoven poly(lactic-*co*-glycolic acid) PLGA sheets assembled to valvular shape by needle punching	•*In-vitro* dynamic for 1 month •*In-vivo* in sheep up to 20 weeks		•9 early dead out of 19 animals, mainly due operatory and anesthesia complications •Trivial regurgitation at implant, increased with time to sever in some animals •Leaflets immobilization, and dimensional decrease at 10 weeks •Acquired native conduit curvature •Conduit diameter remained stable	(158)
•Sheep BM-MSCs	•Nonwoven PGA:PLLA 50:50 scaffold sewn around a plastic frame •Basic fibroblast growth factor (bFGF) and ascorbic acid-2-phosphate (AA2P) addition	•*In-vitro* static up to 3 weeks •*In-vitro* dynamic (pulsatile flow combined with flexural stimulus–CFF) up to 3 weeks	•Higher collagen I & III for bFGF/AA2P samples •Collagen fibers alignment after 6 weeks static •4-fold increase in collagen production due to dynamic conditions •3-fold increase in collagen production due to bFGF/AA2P addition •CFF conditioning increased collagen production by 12% in bFGF/AA2P samples	•Enhanced tissue formation for the bFGF/AA2P samples vs. the basal medium ones •Loos of cellularity (DNA content) with time, possibly due to inefficient cell attachment on absorbed serum proteins, but dynamic condition could keep the DNA level steady •Decreasing of GAG production over time	(159)
•For the scaffold production: ovine vascular-derived cells •Final cellularization: BM-MSCs	•Nonwoven PGA mesh coated with 1.75 % solution of P4HB integrated in nitinol stent	•*In-vitro* static up to 3 days from MSC seeding	•Abundant amount of crosslinked collagen •99% of DNA reduction after decellularization •Significant reduction of glycosaminoglycans •No reduction of hydroxyproline •No differences between radial and circumferential direction in the tensile tests	•Decellularization of vascular derived cells and MSCs seeding hampered leaflets retraction •Valve dynamic performance improved by decellularization •18 months storage did not affect histological appearance and mechanical properties	(160)
•Rat BM-MSCs	•Decellularized rat aortic valvular conduit w/w or w/o a multilayer of heparin + SDF-1α		•Treated scaffold: uniform luminal vWF+, CD34+ under the endothelium (EPCs), large amount of α-SMA in the adventitia, few mononuclear cells and macrophages infiltration (CD45+ and CD68+) •Untreated scaffold: sporadic vWF+, no CD34 detected, large amount of α-SMA in the adventitia, few mononuclear cells and macrophages infiltration (CD45+ and CD68+)	•100% patency and no evidence of stenosis at 4 weeks •Treated scaffold: continuous endothelium, reduced platelet adhesion •Untreated scaffold: no endothelialization	(152)
•Human BM-MSCs	•PGA/P4HB composite	•*In-vitro* static to *in-vitro* dynamic (pulse duplicator) conditioning	•H&E showed cellular tissue organized in a layered fashion, with a dense outer layer and lesser cellularity in the deeper portions after 14 days in the pulse duplicator. •Positive staining for collagen types I, III, α-SMA, and vimentin. •No positive staining for desmin, collagen types II and IV. •Myofibroblasts-like actin/myosin filaments, collagen fibrils and elastin fiber networks	•All leaflets were intact, mobile, pliable; and the constructs were competent during valve closure •Static controls showed a loose, less organized tissue formation with irregular cellular ingrowth •*In-vitro* dynamic conditioned valves comparable to those of native human semilunar valve. Static controls significantly weaker •Confluent endothelial layer in the dynamic *in-vitro* conditioned samples, static samples with non-confluent endothelial layer •Not achieved typical three-layered structure	(150)
•Human putative BM-MSCs	•Decellularized porcine aortic valve homograft	•*In-vitro* static	•Homograft seeded cells were α-SMA, vimentin positive and desmin negative •Seeded cells expressed osteopontin, osteonectin, alkaline phosphatases •Leaflets shrank by 85%	•These cells have good potential for tissue engineering because of their plasticity •Osteogenic markers on reseeded cells were attributed to the prolonged static culture without the proper mechanical cues •Good migration rate of the reseeded cells	(161)
•Human mesenchymal progenitors from prenatal chorionic villus specimens •Human EPCs from postnatal umbilical cord blood	•Sheets of nonwoven mesh PGA dip coated in a 1% (w/v) solution of P4HB shaped in valve fashion	•*In-vitro* dynamic up to 4 weeks	•Markers expression (vimentin, α-SMA positive, desmin negative) matching the native tissue •TEHV presented collagen on the surfaces and GAG in the interstitium whereas native tissue had more homogeneous distribution •GAGs amount comparable, DNA 68%, and hydroxyproline 14% of the native tissue •Better mechanical performance of mechanically stimulated constructs	•Tissue organization comparable with native neonatal valves •Good ingrowth of mesenchymal progenitors from prenatal chorionic villus and complete coverage of EPCs	(162)
•Human MSCs	•Decellularized ovine aortic valves	•Group A: static •*In-vitro* dynamic (pulse duplicator) conditioning with different pressure patterns: •Group B: cyclic negative pressure up to 72 h •Group C: cyclic negative pressure up to 72h, cyclic positive pressure up to 10 days	•Compared to group A (static conditioning): •Group B overexpressed ACTA2 and HSP47/ SERPINH2 (VICs), RUNX2 (osteoblast marker), MKI67 (proliferation marker), and BAX (apoptosis marker). Downregulation of ACAN (chondrocytes marker) •Group C overexpressed CD90, CD105, and CD29 (MSC marker); and BGLAP (osteoblast marker) in addition to those of group B •vWF negative in all groups	•MSCs infiltration into the leaflets •Mechanical behavior more closely resembled that of the cryopreserved leaflet than that of the decellularized leaflet	(144)
**EPCs**					
•Autologous EPCs from ovine peripheral blood •Ovine valve-derived ECs	•Sheets of non-woven mesh PGA dip coated in a 1% (w/v) solution of P4HB	•*In-vitro* static	•VEGF exposed ECs and EPCs have enhanced proliferation •TGF-β1 induces trans-differentiation to mesenchymal phenotype (α-SMA+) •Valve-derived ECs have traces of spontaneous trans-differentiation, which attunes them with the trans-differentiation during valvulogenesis	•Seeded cells respond (proliferate) to VEGF (valve-derived ECs have attenuated response) •TGF-β1 induces trans-differentiation to mesenchymal phenotype	(163)
•Autologous EPCs from ovine peripheral blood •Ovine vascular ECs •Ovine SMCs as control	•Sheets of nonwoven mesh PGA dip coated in a 1% (w/v) solution of P4HB sewed to form valvular conduit shape	•*In-vitro* dynamic up to 21 days	•CD31 and VEGF-R2 positive cells on the luminal surface •α-SMA+ into the interstitium, sign of trans-differentiation (EMT) •vWF positive cells throughout the valve conduit •Decrease of DNA and collagen content at 21 days compared with 7 days •Increased S-GAG content at 21 days •Interstitium with less ECM and more scaffold leftovers •Metalloproteinases (MMPs) and their inhibitors (TIMPs) concentration decreased with time, allowing ECM build-up	•Cellular ingrowth throughout the scaffold •TE valve marker expression similar to native valve, with mesenchymal profile in the interstitium •Seeded scaffolds have 41-fold greater stiffness than the unseeded constructs •CD31+ and α-SMA+ cells have native-like spatial distribution •Suspected lack of nutrient diffusion inside the scaffold •Arterial-derived cells seeded scaffold showed loss of structural integrity •Many of the hallmarks of the initial stage of valvulogenesis are seen in this study •The construct underwent a developmental event	(164)
•Mononuclear bone marrow cells and peripheral blood EPCs cultured in fibroblast and endothelial inducing media respectively •Autologous jugular vein myofibroblasts + carotid artery ECs as a comparison	•P(L,DL)LA (Poly(L-lactide-co-D,L-lactide)) multifilament fibers using a 3-dimensional valve-shaped cast and thermal fixation. Surfaces coated with P(L,DL)LA	•*In-vitro* static sequential seeding; following, *in-vitro* dynamic; and finally, *in-vivo* in sheep model up to 8 weeks	•eNOS positive on surface, α-SMA expression detected on the surface and in the interstitium. •Good GAG deposition •No detectable amount of elastin	•Leaflets thickening, lowering pressure gradient with time, and minimal regurgitation •No indication of immune reaction •Discontinuous endothelial with bits of fibrous material deposition •Absence of elastin might have caused reduced pliability	(151)
•Autologous sheep EPCs	•Decellularize porcine pulmonary valves	•*In-vitro* static seeding; then, *in-vitro* dynamic conditioning; and finally, *in-vivo* in sheep model up to 3 months	•EPCs scaffold were let express ECs markers in EGM media before scaffold seeding •MMP9 was found in CD133-conjugated scaffolds	•CD133-conjugated scaffold were repopulated better *in-vivo* than already seed scaffolds	(165)
•Human EPCs from umbilical cord blood •Human Wharton's jelly-derived myofibroblasts	•Sheets of nonwoven mesh PGA dip coated in a 1% (w/v) solution of P4HB and attached to ring-shaped supports	•*In-vitro* dynamic with static as control	•Growth factors addition resulted: in GAGs amount comparable with native tissue, DNA 65% of native tissue, hydroxyproline 16% of native tissue •No traces of elastin	•Dense cell coverage and leaflet pliable •Phenotype differentiation and tissue organization just in biochemical stimulated construct •Mechanical stimulation enhanced scaffold mechanical properties •Worthon's jelly-derived myofibroblasts might have enhanced tissue ECM production and organization	(166)
•Human EPCs from venous blood	•Decellularized porcine aortic, valve heparin and VEGF coated	•*In-vitro* static up to 48h		•Coated valves had higher number of adherent cells •Higher proliferation rate on the coated valves •Higher migration rate on the coated valves	(167)
•Autologous human EPCs from peripheral blood (mononuclear fraction)	•Decellularized human pulmonary valves from cadaver	•*In-vivo* human trial on 2 pediatric patients, follow-up to 42 months	•Peripheral blood mononuclear cells were expressing CD31, vWF, VEGF-R2	•No arrythmia or any other problems in the follow-up •Somatic growth of both the valves and the patients •Trivial regurgitation after 1 year •No signs of malformation •Normal transvalvular gradient	(168)
•Human mesenchymal progenitors from prenatal chorionic villus specimens •Human EPCs from postnatal umbilical cord blood	•Sheets of nonwoven mesh PGA dip coated in a 1% (w/v) solution of P4HB shaped in valve fashion	•*In-vitro* dynamic up to 4 weeks	•Markers expression (vimentin, α-SMA positive, desmin negative) matching the native tissue •TEHV presented collagen on the surfaces and GAG in the interstitium whereas native tissue had more homogeneous distribution •GAGs amount comparable, DNA 68%, and hydroxyproline 14% of the native tissue •Better mechanical performance of mechanical stimulated constructs	•Tissue organization comparable with native neonatal valves •Good ingrowth of mesenchymal progenitors from prenatal chorionic villus and complete coverage of EPCs	(162)
•Human amniotic fluid mononuclear cells split in CD133+/- •Human CD133+ to differentiate in ECs (VEGF, hFGF, R-3-IGF-1) •Human CD133- to differentiate into mesenchymal cells (hFGF, R-3-IGF-1, GA-1000, ascorbic acid,20% fetal bovine serum)	•Sheets of nonwoven mesh PGA dip coated in a 1% (w/v) solution of P4HB shaped in valve fashion	•Sequential seeding, followed by *in-vitro* dynamic culture up to 28 days	•TEHV presented collagen on the surfaces and GAG in the interstitium •DNA amount comparable, GAG 80%, and hydroxyproline 5% of the native tissue	•Valve had homogeneous thickness •Mechanical properties did not reach the physiological values, probably due to the low amount of hydroxyproline	(169)
•Human EPCs from cord blood	•Porcine decellularized heart valves functionalized with RGD, VEGF, PEG	•*In-vitro*		•Functionalized scaffolds had enhanced early attachment •Functionalized scaffold favors the proliferation, a cell spread cell morphology, and a complete endothelialization	(170)
**iPSCs**					
•Human skin fibroblast reprogrammed into iPSCs, then, differentiated into MSCs	•Decellularized human pulmonary valve	•*In-vitro* up to 14 days	•iPSCs-MSCs have twice the proliferation rate of BM-MSCs •iPSCs-MSCs on surface expressed α-SMA, whereas iPSCs-MSCs in the interstitium did not	•iPSCs-MSCs produced ECM (glycosaminoglycans and collagen) •iPSCs-MSCs shown several similarities to VICs	(171)
•Human iPSCs differentiated into MSCs	•PEGDA coated dishes and 3D PEGDA hydrogels	•*In-vitro* up to 20 days	•3D PEGDA cultured iMSCs had similar α-SMA expression to VICs •iMSCs had higher level of collagen expression •3D PEGDA cultured iMSCs had lower expression of calponin compared to VICs	•3D PEGDA cultured iMSCs had similar α-SMA expression to VICs	(172)
**VICs and VECs**					
•VICs from porcine aortic valve	•Fibronectin or collagen or heparin coated wells	•*In-vitro*		•VICs seeded on fibronectin coated wells in presence of TGF-β1 are activated and stimulated to produce stress fibers and express α-SMA •Heparin increases TGF-b1 production in VIC monolayer culture	(173)
•VICs from porcine aortic valve	•Porcine decellularized leaflets	•*In-vitro*		•5% serum for 12h improved the VICs proliferation on the scaffolds	(174)
•VICs from porcine aortic valve	•Functionalized PEG hydrogel: four-arm poly(ethylene glycol) (PEG) chains connected with enzymatically degradable peptides and RGD	•*In-vitro*	•RGD increase the spreading •TGF-β1 increases α-SMA and collagen I expression	•Stiffer surfaces enhanced myofibroblastic activity of VICs •MMP biodegradable hydrogels allowed migration and cell spreading, higher with less crosslinked hydrogels •Good proliferation, 7 days of doubling time	(175)
•VICs from porcine aortic valve	•Different ratios Polyacrylamide/bisacrylamide coated wells to have different substrate stiffness	•*In-vitro*	•TGF-β1 did not impact cell density or morphology. On the other hand, it did influenced cell spreading, and α-SMA expression •Bigger cells expressed more α-SMA	•Higher substrate stiffness increased cell spreading and influenced morphology (cytoskeletal organization and focal adhesion arrangement)	(84)
•Autologous EPCs from ovine peripheral blood •Ovine valve-derived ECs	•Sheets of nonwoven PGA mesh dip coated in a 1% (w/v) solution of P4HB	•*In-vitro*	•VEGF-exposed ECs and EPCs have enhanced proliferation •TGF-β_1_ induces trans-differentiation to mesenchymal phenotype (α-SMA expression) •Valve-derived ECs have traces of spontaneous trans-differentiation, which attunes with the trans-differentiation during valvulogenesis	•Seeded cells respond (proliferate) to VEGF (valve-derived ECs have attenuated response) •TGF-β_1_ induces trans-differentiation to mesenchymal phenotype	(163)
•Model 1: porcine aortic VICs •Model 2: porcine aortic VICs + lining of porcine aortic VECs	•Collagen gel	•*In-vitro* dynamic in a parallel plate flow chamber up to 96 h at shear stress of 20 dyne/cm^2^	•Change of VECs alignment under the flow •VICs in Model 2 migrated toward the surface, perhaps due to the addition diffusion barrier made by the VECs •Model 2 had lost cells, Model 1 no •Model 2 increased the protein content under flow •Vimentin is maintained in both models •α-SMA was less in Model 2 •VECs maintained their phenotype in Model 2	•VECs downregulates α-SMA in VICs •Model 1 static proliferation can resemble the wound healing process (no VECs)	(73)
•Sheep VICs •Sheep VECs •Sheep CAECs •Sheep EPCs from peripheral blood •Human cord blood EPCs (hcbEPCs) •Human dermal microvascular ECs (hDMECs)	•Flasks	•*In-vitro*	•VECs in TGF-β_1_ rich media got mesenchymal-like phenotype (α-SMA upregulated, CD31 downregulated) •Less than above VECs samples were able to express osteogenic markers in differentiating media	•No endothelial cell type, apart from valvular was able to express osteogenic markers •VECs on leaflets expressed osteocalcin after mechanical stretching	(72)
•Porcine aortic VECs	•Silicone with different stiffness levels	•*In-vitro* with TGF-β_1_subministration to induce EMT	•Stiffer substrates induced EMT in presence of TGF-β_1_ shown by spindle-like morphology, VE-cadherin downregulation, and α-SMA upregulation •B-catenin inhibition reduces α-SMA upregulation	•Stiffer substrates induced EMT in presence of TGF-β_1_	(85)
•Both human and porcine VICs	•Electrospun polyglycerol sebacate (PGS) and PCL blends	•*In-vitro*	•PGS decrease the contact angle and enhance cell attachment and spreading when blended with PCL •PGS makes the scaffold quicker to degrade •No elastin synthesis	•Slower spreading in PCL scaffolds •VICs produce more ECM in PGS-PCL blends	(176)
**ECs** + **MYOFIBROBLASTS/FIBROBLASTS/VSMCs**				
•Autologous jugular vein myofibroblasts •Carotid artery ECs	•Poly (glycolic acid)-(PGA)-Poly-4-hydroxybutyrate(P4HB) stented scaffold	•*In-vitro* dynamic; then, *in-*vivo in sheep model up to 8 weeks	•DNA content 49 ± 24%, GAG content 39 ± 9%, hydroxyproline content 15 ± 6% that of native t. at 4 weeks •DNA content 44 ± 18%, GAG content 39 ± 6%, hydroxyproline content 18 ± 3% that of native t. at 8 weeks	•Proper opening and closing behavior, minimal regurgitation in 2 animals •Leaflet thickening, hosting wall integration •Cell attachment and ingrowth •Cellular tissue formation and abundant amounts of collagen in the leaflets, no elastin detected, incomplete ECs layer	(151)
•Autologous Carotid myofibroblasts •Autologous Carotid ECs	•PGA/P4HB composite	•*In-vitro* static; then, *in-vitro* dynamic (pulse duplicator); finally, *in-vivo* in sheep model up to 20 weeks	•Bioreactor conditioning increased organization and layering of the leaflet structure. •DNA content (150% that of native t.), collagen (180% that of native t.), GAGs (140% that of native t.), limited elastin traces by 6 weeks. ECs were CD31, vWF positive; myofibroblasts were α-SMA positive	•No evidence of thrombus, stenosis, or aneurysm formation up to 20 weeks. •Central pulmonary regurgitation (mild to moderate) •Increase of the inner diameter of the valve constructs •Mechanical properties at 20 weeks were almost indistinguishable from those of native valve tissue	(149)
•Autologous iliac crest bone marrow myofibroblast-like cells •Peripheral blood endothelial progenitors	•Multi-layered P(L,DL) LA (Poly(L-lactide-co-D,L lactide)) stented scaffold	•*In-vitro* dynamic; then, in sheep model up to 4 weeks	•DNA content 86 ± 54%, GAG content 150 ± 11%, hydroxyproline content 26 ± 6% that of native t. at 4 weeks •Cellular tissue formation and abundant amounts of collagen in the leaflets, no elastin detected, incomplete ECs layer •Cells staining positive for α-SMA were identified mainly in the wall of the explanted valves, but also in the middle of the leaflets •On the leaflet surfaces, endothelial nitric oxide synthase (eNOS) expression detected	•Proper opening and closing behavior, minimal regurgitation in 2 animals •Leaflet thickening, hosting wall integration •Cell attachment and ingrowth	(149)
•Ovine carotid artery ECs and SMCs •Juvenile sheep bone-marrow derived CD133+ cells	•Decellularized stented hybrid ovine small intestine submucosa/ porcine pulmonary valve	•*In-vitro* static up to 3 days; then, *in-vitro* dynamic (pulsatile flow bioreactor); finally, *in-vivo* in sheep model up to 3 months	•A confluent monolayer was demonstrated by CD31-staining in both groups. •Immunohistochemistry revealed strong expression of αSMA and an ingrowth into the leaflets in the two groups (higher for CD133+ cells) •CD3, CD20, CD45, and CD68 staining confirmed no signs of inflammation in all animals in group 2, whereas in group 1, small amounts of inflammatory tissue were detected in all animals	•Valve good opening and closing characteristics in both groups, no or minimal regurgitation, and a low transvalvular gradient (higher in the ECs/SMCs group) •Smooth surfaces without any thrombus formation •Mild to moderate calcifications in the annular region of the valve stents in group 1, microcalcifications were detected in one of five animals in group 2	(155)
•Human foreskin fibroblasts (hFFs) •Human adipose derived stem cells (hADSCs) endothelial differentiated (hCFECs)	•Decellularized porcine pulmonary valve	•*In-vitro* up to 6 days		•hFFs and hCFECs were able to colonize the scaffolds and penetrated at 6 days •hCFECs deployed a layer on top	(177)
•Human vascular ECs and FBs from saphenous vein	•Polyurethane PU sheets made with spraying technique	•*In-vitro* sequential static seeding; then, dynamic culture	•Increase of cellular adhesion molecules •Increase of Collagen 4, VE-Cadherin, and Fibronectin expression after dynamic culture •Increase of inflammatory cytokines but the gene expression did not confirm that	•Good endothelial lining orientated with the flow •Good FBs layer	(143)
•Aortic SMCs •Aortic adventitial fibroblast/myofibroblast •Umbilical vascular endothelial cells (hVECs) In a tri-layered fashion	•Nitinol mesh-enclosed leaflets with cell layers embedded in collagen	•*In-vitro* static; then, *in-vitro* dynamic (pulsatile flow)	•SMCs degraded and contracted the collagen but then ECs stoped their action	•The leaflets had a correct functioning in the bioreactor	• (178)

*ECs, endothelial cells; PGA, polyglycolic-acid; PLLA, poly-l-lactic acid; P4HB, poly-4-hydroxybutyrate; PCL, polycaprolactone; t, tissue; GAGs, glycosaminoglycans; vWF, von Willebrand factor; H&E, haematoxylin eosin; BM, bone marrow; MSCs, mesenchymal stromal cells; PB, peripheral blood; EPCs, endothelial progenitor cells; α-SMA, alpha-smooth muscle actin; eNOS, endothelial Nitric Oxide Synthase; MNCs, mononuclear cells; SMCs, smooth muscle cells; CAECs, coronary artery endothelial cells; SDF-1α, stromal cell-derived factor-1α; VICs, valve interstitial cells; ACTA2, gene codifying for smooth muscle alpha (α)-2 actin; HSP47/ SERPINH2, gene codifying for Serine (Or Cysteine) Proteinase Inhibitor, Clade H (Heat Shock Protein 47), Member 2; MKI67, gene codifying for marker of proliferation Ki-67; BAX; gene codifying for BCL2 Associated X, Apoptosis Regulator; ACAN, gene codifying for Aggrecan/ Chondroitin Sulphate Proteoglycan Core Protein 1; BGLAP, gene codifying for bone Gla protein/osteocalcin; w/w, with; w/o, without; SEM, scanning electron microscope*.

Two main scopes are followed for graft repopulation: (i) to recreate the internal biologic environment of a valve and (ii) to provide them with an EC coverage. Short-term follow-up studies in sheep and primates showed the potential advantage of repopulating the core of scaffolds/grafts with VICs or MSCs (156, 179–181). However, other aspects such as the prone differentiation into myofibroblast or osteoblast phenotypes must be considered and is discussed below for the main cell sources explored so far.

### Mesenchymal stem cells (MSC)

MSC with different origins seems to be a consistent choice for the TEHVs cellularization since the VIC represents a heterogenous population of cells sharing a mesenchymal ancestor. Moreover, the VIC shares phenotypic commonalities with VSMC and fibroblasts, that could be achieved by MSC differentiation as confirmed by antigenic expression (21, 22, 156, 157). In addition, MSCs are easy to be harvested and expanded *in vitro*, and there are multiple tissue sources (e.g., bone marrow, adipose tissue, peripheral blood, umbilical cord blood, umbilical cord, and placenta or amniotic fluid). Both animal and human studies support the immunoprivileged state of the MSC and evidences their unique immunomodulatory characteristics. Accordingly, the MSC is nowadays the preferred cell of choice for *in vitro* TEHV and the several studies in animal models account for that (147, 182). Since MSCs are progenitor stem cells able to differentiate in all the valvular cell phenotypes, they can overcome the issue of primary cells harvested from old and sick patients (147). Finally, unlike other stem cells, MSCs do not develop teratomas and there are not ethical concerns as for the embryonic stem cells (ESCs) (183).

MSC-bioengineered valves differentiated through conditioning in biomimetic and dynamic environments have shown physiologic profiles in terms of ECM composition (e.g., higher amounts of collagen type I and III), mechanical properties and VIC/myofibroblast markers expression (147, 183). These studies have also shown the influence of chemical, flow, and flexural stimuli on the cell phenotype expression and synthesis capabilities, also demonstrating an enhanced outcome of their synergic action. Moreover, MSC preserve their phenotype plasticity, being able to express endothelial or mesenchymal markers in response to different biochemical and mechanical stimuli (157). No evidence of glycosaminoglycans synthesis has been demonstrated by MSCs, but that issue could be circumvented by an additional stimulation with concomitant insulin and hypoxic conditioning (184). *In vivo* experiments performed on rat, sheep, and canine models have confirmed the positive *in vitro* outcomes. MSCs differentiation and different secretion of ECM components were mirroring the native structure (156). Cell labeling of implanted cells has also suggest their active collaboration in tissue regeneration (152, 153). However, performance issues, such as regurgitation and leaflets mobility restriction, have been found in some MSC-bioengineered substitutes (158).

Animal model, mainly pig and sheep, can give some help to test the valve ability to withstand some aspect of the immune reaction and calcification. Sheep model are preferred because of their valve anatomy similarity with humans, and the slower growth pace compared to the pigs. Moreover, juvenile sheep models represent the worst-case scenario to evaluate calcification because of the high level of calcium and phosphorous in the serum. However, they do not consider all the peculiarity of the human immune system. For instance, sheep have reduced platelet activity than the humans (185). Therefore, many *in vivo* studies ended up in failures when translated to clinical practice (186, 187). Comparison studies attempted to determine the superiority of available cells products, in particular, MSCs vs. other cell types, such as BM-mononuclear cells (MNCs) or CD133^+^ aortic-derived cells (144, 154, 188). An interesting report from Vincentelli et al. (154) compared the efficacy of MSC- or MNC-engineered TEHVs implanted in lambs. Both cell groups promoted the re-endothelization of the TEHV through recruitment by the recipient's ECs after 4 months implantation. However, MNC-seeded valves caused leaflet thickening, retraction, inflammation and calcification, while the MSC-seeded valves displayed a αSMA^+^ cellularization with no signs of calcification.

Controversial results have been observed in humans and the therapeutic use of MSCs (188). Modest or null benefit has been documented in clinical trials using BM-MSCs (189, 190). In the context of VHD, experiments are mostly limited to *in vitro*, static or dynamic, TEHV cellularization (185, 186). The experimental results of human *in vitro* studies are aligned with the animal-derived MSCs *in vitro* models. Dynamic culture enhanced the construct mechanical properties, which were comparable to the native valves; tissue formation and organization; endothelialization; and native-like markers patterns (150). Different mechanical stimuli, or different intensity of them, promote several MSC behaviors such as migration or differentiation (144). For example, media enriched with VEGF and high shear stress leads to endothelial phenotype differentiation (191, 192). Concerns also surround the stability of the acquired phenotypes and the potential unwanted fibrotic overgrowth causing retraction and regurgitation of the TEHV. In fact, osteogenic markers (such as alkaline phosphatases, osteopontin, and osteonectin) have been found expressed in the implanted graft, suggesting the susceptibility to prosthetic valve calcification (132, 147). Pro-osteogenic cells may influence resident VICs to acquire similar properties, thus raising concerns about their transplantation into pro-calcifying environments.

### Endothelial progenitor cells (EPCs)

Similarly to the MSCs, the endothelial progenitor cells (EPCs) have broaden differentiation potential and can be supplied by non-surgical procedures, since they can be isolated from peripheral and umbilical cord blood (147). EPCs are particularly interesting because of their capability to differentiate into EC and to contribute to vascular regeneration and development as well as to neovascularization processes after limb or myocardial ischemia (193). Moreover, EPCs can undergo EMT processes to acquire a VIC-like phenotype under determinate stimuli (e.g., growth factors such as TGFβ1 or mechanical conditioning) and therefore offering the possibility of a complete valve regeneration with a single cell type (163, 194, 195). However, EPCs may also contribute to different pathological stages including cancer and diabetes (196, 197) and the ideal antigenic profile remains controversial (165).

*In vitro* and *in vivo* experiments have demonstrated the ability of the EPCs to colonize the whole TEHV. Importantly, EPCs express both endothelial and mesenchymal lineage markers (CD31 and α-SMA, respectively), spatially arranged in a native-like fashion with a concomitant expression of MMPs and TIMPs, suggesting an active remodeling which recapitulates a developmental-like process (198). The result is a higher mechanical performance than the one achieved by other cell sources (164). No calcification or thrombi were noticed in all the reported studies. Furthermore, EPCs have been implanted on valve leaflets in animal models leading to a reduced infection and graft failure (199). However, the lack of leaflet pliability and discontinuous endothelialization raise concerns about the EPC-based bioengineered devices (151).

*In vitro* studies conducted on human-derived EPCs show similar results to those find *in vitro* and *in vivo* using animal sources. Human EPCs derived from umbilical cord blood have been co-seeded with Wharton's Jelly-derived myofibroblasts. Biochemical and mechanical stimulations are also necessary to promote the desired native-like mechanical organization, phenotype determination, and functionalization (166, 167, 170). Human EPCs harvested from umbilical cord blood have been also combined with prenatally harvested chorionic villus-derived MSCs to provide a tissue engineered prosthesis for pediatric patients. A good phenotype and mechanical properties of the resulting prosthetic valve was achieved (162).

Clinical studies of re-endothelialization have confirmed the feasibility of correcting pulmonary valve defects using allografts engineered with vein-derived autologous ECs or EPCs (200). To the best of our knowledge, the only one human *in vivo* study took place in Republic of Moldova in 2002 and was published on Circulation in 2006. Two pediatric patients affected by tetralogy of Fallot, underwent a pulmonary valve replacement with decellularized human pulmonary valves which have been repopulated by autologous MNCs from their peripheral blood. After valve recellularization, the cells were characterized as EPCs. Throughout the follow-up duration (3.5 years), the patients recovered well. Themselves and their prosthetic valves had a somatic growth, and there was no complication whatsoever. Only a trivial regurgitation was reported (168).

### Induced pluripotent stem cells (IPSC)

In some cases, adult stem cells are not enough proliferative due to diseases or patient old age. A good alternative might be the iPSC, which are autologous reprogrammed fibroblasts, able to differentiate in MSCs and ECs. Using this cell type, ethical or need for compatible stem cells issues are avoided. Simpson et al. managed to reprogram skin fibroblast into iPSC, and then produce iPSC -derived MSCs (iPSC -MSCs) and iPSC -derived ECs (iPSC -ECs). iPSC -MSCs were seeded on decellularized human pulmonary valves, resulting in valve repopulation and ECM production (171). Compared with MSCs, the iPSC -MSCs have higher proliferation potential and have some expression pattern similarities with VICs (172). However, there are still safety concerns about IPCSs. Recent reports have emphasized the pitfalls of iPSC technology, including the potential for genetic and epigenetic abnormalities, tumorigenicity, and immunogenicity (148).

### Native cell types: VIC and VEC to bioengineer TEHV

TEHV seeded with human mitral or aortic VICs can generate a valvular tissue with mechanical properties similar to the naive human aortic valve (176) while retaining native antigenic expression (201, 202). Similar results have been reported for human VICs isolated from sclerotic valves (146). However, several models using native VICs and VECs to produce *in vitro* TEHV aim to model the pathological development of prosthetic degeneration, calcification and fibrosis by mimicking native-like environments rather than to design prosthetic solutions (203, 204). Other studies have used native resident cells to establish tissue engineering and cell culture protocols (147, 176, 205). For instance, VICs seeded on heparin-coated wells in presence of TGF-β1 undergo to activation into myofibroblast phenotypes with enhanced synthetic and contractile activity, producing stress fibers and expressing α-SMA, all of them typical markers of active VICs (173). Mechanical properties of the scaffolds are also tested on native resident cells to better understand the mechanobiology of the VIC. Stiffer surfaces enhance their myofibroblastic activity, their density, and spreading (84). Conducting those studies is especially relevant to develop methods capable to obtain a temporary myofibroblast phenotype. Myofibroblasts are known to exert wound healing function but its persistence may promote fibrosis and calcification causing prosthetic valve disease (81, 83) as well as prosthetic retraction and regurgitation due to an excessive contractibility (206).

*A priori*, more interesting for therapeutic applications would be the VEC given the phenotypic peculiarities mentioned above. Those unique properties allow the VEC to provide an antithrombogenic surface, replenish the VIC population toward EMT processes and regulate VIC phenotype, in response to mechanical and biochemical stimuli. Indeed, Butcher et al. described the capability of the VEC to maintain quiescence of bioengineered VICs (73). However, difficult harvest and their degeneration can lead to valve failure due to neo-vascularization, infiltration of inflammatory agents, or lipid deposition, amongst the many (207). In addition, VECs undergoing to EMT can express osteogenic markers, not reported in other EC populations (72, 85). Although VECs can trigger valve dysfunction, providing an endothelial lining is a main concern when designing TEHV. That justify the use of primary endothelial cells (ECs) or EPCs in many studies (147).

### Other native resident cells: ECs, myofibroblasts/fibroblasts/VSMC

A way to attempt to reproduce the valvular structure is through using cell types whose lineage is close to VICs and VECs including ECs, fibroblasts (FBs) and VSCM. Sequential seeding of FBs and human adipose-MSC-derived endothelial cells were able to colonize and penetrate into animal decellularized heart valves (177). However, activation of FBs and VSMC into myofibroblasts has been also reported leading to expression of cytokines, and scaffold degradation and contraction (143, 178). Incorporation of ECs lining seems to stop it (178). Those approaches are getting us close to understand the mechanisms to switch off and on undesired phenotypes by controlling the mechanical and biochemical signals of the designed TEHV (208). In line with *in vitro* results, TEHV bioengineered with myofibroblasts and ECs in sheep for 8–20 weeks was associated with leaflet thickening and moderate regurgitation (149, 151). Incipient calcification and regurgitation have been also found in BM-derived SMC valve substitutes implanted in sheep (155).

The utility of using native resident cells stands also in establishing the suitability of scaffolds designated for *in situ* TEHV and their capability to accommodate the recruitment of resident cells and a proper phenotype.

### Alternative cell sources

There are other cell products with potential application in the *in vitro* TEHV with minor or not assessment so far. For example, two fractions have been differentially characterized among the progenitor cells isolated from the amniotic fluid. It has been shown that CD133^+^ fraction of the mononuclear cells in the amniotic fluid can acquire endothelial phenotype, whereas the CD133^−^ fraction can differentiate into myofibroblast-like cells. Those cells are especially relevant for the preparation of cellularized valves before birth (169). An unexplored alternative for *in vitro* TEHV in the adult is the suitability of heterogeneous perivascular stem/progenitor cells described in the vascular niche by our group and others and considered native ancestors of heterogenous MSCs (179, 209). The therapeutic potential of those perivascular cells in the cardiovascular regenerative medicine has been already demonstrated (210–212). Moreover, adventitial perivascular progenitor cells (APC) derived from cardiac surgery saphenous vein leftovers have properties, which make them a potential candidate for regenerative medicine (145), including the suitability for cellularization of xenografts (213) and application into myocardial ischemic models (214–216). The latter has shown the superiority of the APC to keep their specialized function upon implantation without acquiring undesired phenotypes (214). Importantly, intramyocardially transplanted APCs did not induce calcification, in contrast with BM-MSCs. It remains unclear if these properties are peculiar to APCs. *In vivo* and *in vitro* studies demonstrated the capability of other pericytes to contribute to the pathogenesis of vascular calcification toward osteogenesis and angiogenesis promotion from the adventitial *vasa vasorum* and the intimal layer (217). However, no intact perivascular coat has been described yet around the new vessels irrigating the growing of the advanced plaque-like tissue (87) and BM-MSC-derived endothelial cells and adventitial Sca1^+^ cells, rather than derived from adventitial *vasa vasorum*, have been described in association with atheromatous plaque progression (218, 219). Further evidences are needed to state that the APC is a cell of choice for *in vitro* TEHV. Adding new cell sources may bear the risk of adding more approaches to the several techniques and approaches found in the literature.

## Additional consideration to choose the proper cell type for *in vitro* TEHV

In order to improve graft durability, additional aspects must be considered. (A) *Cell-graft interactions*. These features are inherent to the cells, but also depend on proper interactions between the “right cell” and “right prosthesis.” For instance, VEGF significantly inhibits the formation of calcium nodules when ovine VICs are grown on collagen, fibronectin, and laminin (97), while may confer osteoblast-like phenotypes using other substrates, suggesting that providing “right” specific ECM and/or growth factors may protect VICs from calcification and degeneration. Combining cells and prostheses already available in a clinical format may provide the means for swift exploitation. Thus it may be advantageous to test them first. (B) *Cell accessibility and scalability*. Tissues that are easily accessible as a source of candidate cell products represent the ideal solution. However, thanks to advances in cardiac imaging, it is now possible to obtain tissue specimens for cardiac cell harvesting with minimally invasive procedures. Additionally, expansion and storage protocols of various cell types are well established, thus allowing potential use of diverse cell populations for TE. (C) *Tissue specificity*. It is thought that progenitor cells and differentiated cells maintain an epigenetic memory of the source tissue. In line with this concept, cardiac progenitor cells, VICs and VECs may represent the logic solution for disease conditions that require reparative cardiomyogenesis or valve replacements. (D) *Paracrine activity*. As discussed above, cells seeded onto the graft/scaffold represent a source of biomolecules, favoring re-endothelialization, new native-like ECM (138). In addition, the presence of cells can decrease the degradation rate of the constituent scaffold ECM resulting in enhanced preservation of its mechanical properties (176) and eventually against prosthetic calcification. (E) *Cell retention on the implanted scaffolds*. This important aspect has not been extensively assessed, because of the difficulties in tracking cells incorporated into the graft. A study investigating TEHVs made by autologous canine BM-MSCs, seeded on allogenic or porcine-derived xenogeneic pulmonary valves demonstrated cell retention of 1 and 3 weeks, respectively (153). It remains uncertain whether the pathologic and pro-calcifying environment found in the aortic wall contiguous to the prosthetic valve implantation site may affect the retention and “right” phenotype preservation of cells used for TEHV and that needs to be studied.

## Conclusions

A wide range of approaches is still being explored in the manufacture of TEVHs, based on established technologies and novel cutting-edge techniques. Due to many patients targeted by TE for substitution of cardiac valves, the financial volume for these technologies/products is substantial. A market forecast for tissue engineered products indicates the total value will surpass $4.8 billion by 2028.

Several publications with promising *in vivo* and *in vitro* results have underestimated the effects of the “minor outcomes” reported and that could lead to valve substitute degeneration in a next generation of the current “*biologic prosthetic valve disease*.” Active native-like ECM deposition and even valvulogenesis-like events must be desirable during the process of valve substitute production, but those must be abolished thereafter to avoid excessive fibrosis, contraction, retraction, degeneration, and calcification of the valve substitute. On this regard, the ideal cell type of choice has yet to be determined and more research is needed to provide the best therapeutic alternative to both adult and congenital VHD. Besides, results from experimental modeling performed with resident native cells seeded on different types of scaffolds, show that scaffold compositions or designs still need to be substantially improved to achieve the correct cell behavior in a diseased environment. Further research on this regard, combined with a better knowledge of the pathology, including the factors triggering myofibroblast phenotype perpetuation, osteoclast recruitment in the calcific valve or the exquisite behavior of the VEC, will significantly contribute to successfully develop valve substitutes. Innovative technologies are required to meet specific, quantitative standards of safety and performance. Similar standards will have to be developed to enable routine clinical use and customized fabrication of TEHVs. While a large number of options have been tested in animal models, more work is warranted before the use of TEHVs can be proposed as a better therapeutic option than available prostheses.

## Author contributions

EJ reviewed the literature, drafted the manuscript, and prepared the figures and tables. MF reviewed the literature, drafted the manuscript, and prepared the tables. GA critically revised the manuscript. PM reviewed the literature, drafted, and critically revised the manuscript. All the authors have approved the final submission of the manuscript.

### Conflict of interest statement

The authors declare that the research was conducted in the absence of any commercial or financial relationships that could be construed as a potential conflict of interest.
